# Directly probing spin dynamics in a molecular magnet with femtosecond time-resolution†
†Electronic supplementary information (ESI) available. See DOI: 10.1039/c6sc01105e


**DOI:** 10.1039/c6sc01105e

**Published:** 2016-08-01

**Authors:** J. O. Johansson, J.-W. Kim, E. Allwright, D. M. Rogers, N. Robertson, J.-Y. Bigot

**Affiliations:** a EaStCHEM , School of Chemistry , University of Edinburgh , David Brewster Road , EH9 3FJ , UK . Email: olof.johansson@ed.ac.uk; b Institut de Physique et Chimie des Matériaux de Strasbourg (IPCMS) , UMR 7504 , CNRS , Université de Strasbourg , BP 43, 23 rue du Loess , 67034 Strasbourg Cedex 02 , France . Email: jean-yves.bigot@ipcms.unistra.fr

## Abstract

We show that a vanadium–chromium Prussian blue analogue, which is a room-temperature molecule-based magnet, displays a fast magnetic response on a femtosecond timescale that is attributed to the super-exchange interaction between the metal ions. These dynamics are obtained from femtosecond Faraday magneto-optical (MO) measurements, performed at 50 and 300 K. Exciting at the ligand-to-metal charge-transfer (LMCT) band results in the formation of the ^2^E excited state on the Cr ion *via* intersystem crossing (ISC) from the ^4^LMCT state in less than 250 fs. Subsequent vibrational relaxation in the ^2^E state occurs on a 0.78 ± 0.05 ps timescale at 50 K and 1.1 ± 0.1 ps at 300 K. The MO measurements can detect the formation of the ^2^E state on the Cr ion from the change in the super-exchange interaction taking place as a result of the corresponding spin flip associated with the formation of the ^2^E state. These results open up a new avenue to study molecular magnets using a powerful method that is capable of directly probing spin dynamics on a sub-picosecond timescale in thin film environments.

## Abstract 2

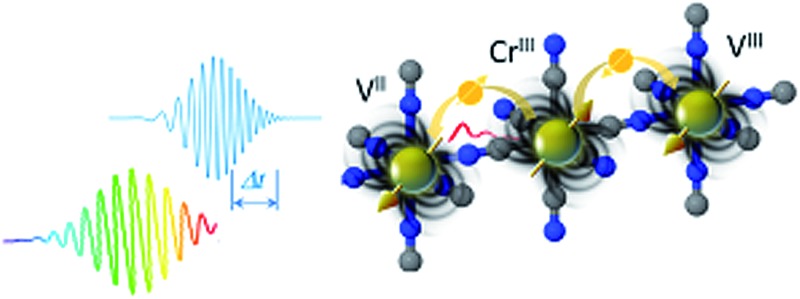
Femtosecond magneto-optical measurements detect the formation of a spin-excited state in the vanadium–chromium Prussian blue analogue, which is a molecule-based magnet.

## Introduction

The ability to optically switch the spin configuration of molecular magnets^1–3^ could contribute to the development of applications such as quantum computers, spintronic devices, and high-capacity information-storage devices. Femtosecond laser pulses currently form the only technology able to function beyond one terahertz (10^12^ Hz), allowing for potentially faster switching than the 10–100 gigahertz capabilities of electronic transistors. To study switching processes, a method is needed that is directly sensitive to the spin state and is fast enough to probe on the sub-picosecond timescale relevant for optical excitation. To this end, ultrafast magneto-optical (MO) techniques,^4,5^ such as Faraday rotation, are the only optical methods capable of directly probing spin dynamics on these timescales, as reported in various magnetic metals,^6^ dielectrics^7^ and nanoparticles.^8^ Applying these techniques to molecular materials therefore offers exciting possibilities since optical spin-manipulation has been achieved in a range of molecule-based magnets^9–16^ and spin-crossover (SCO) systems^17–29^ but so far only X-ray fluorescence using free-electron lasers has provided a direct probe of the sub-ps spin dynamics.^30^

Faraday rotation is closely related to magnetic circular dichroism (MCD) and occurs due to a difference in the index of refraction for left and right circularly polarised light in a magnetised material.^31^ The difference arises because the circular polarisation components interact differently with Zeeman-shifted electronic states whose spin and orbital angular momenta align differently in a magnetic field. Importantly, the Faraday rotation angle is proportional to the sample magnetisation. In time-resolved measurements, the MO signal is obtained by carefully measuring the change in polarisation state of the probe pulse as a function of time delay after exciting the sample with a pump pulse. Ultrafast MO methods have made it possible to untangle spin dynamics from charge and lattice dynamics in ferromagnets after femtosecond laser pulse excitation.^6^ They therefore show great potential to be able to distinguish spin and nuclear dynamics in SCO materials, where high-spin and low-spin states are typically distinguished based on changes in optical spectra^18,23,25,32^ and/or bond-lengths,^21,22,24,33–35^ which are not explicitly sensitive to spin dynamics. The power of ultrafast MO Faraday measurements is that they can give details of magnetisation dynamics on the fs timescale.

In this article, we explore the ultrafast MO and transmission dynamics of thin films of the V^II/III^–Cr^III^ Prussian Blue Analogue (PBA), which was chosen as a model system because it is a room-temperature magnet^36^ with a pronounced static MO response in the visible spectrum.^37,38^ We demonstrate that a change in the spin configuration on the metal ions leads to a sub-picosecond change in the MO signal due to the super-exchange interaction between the metallic ions in the films.

## Experimental

### Materials

**Fig. 1:**
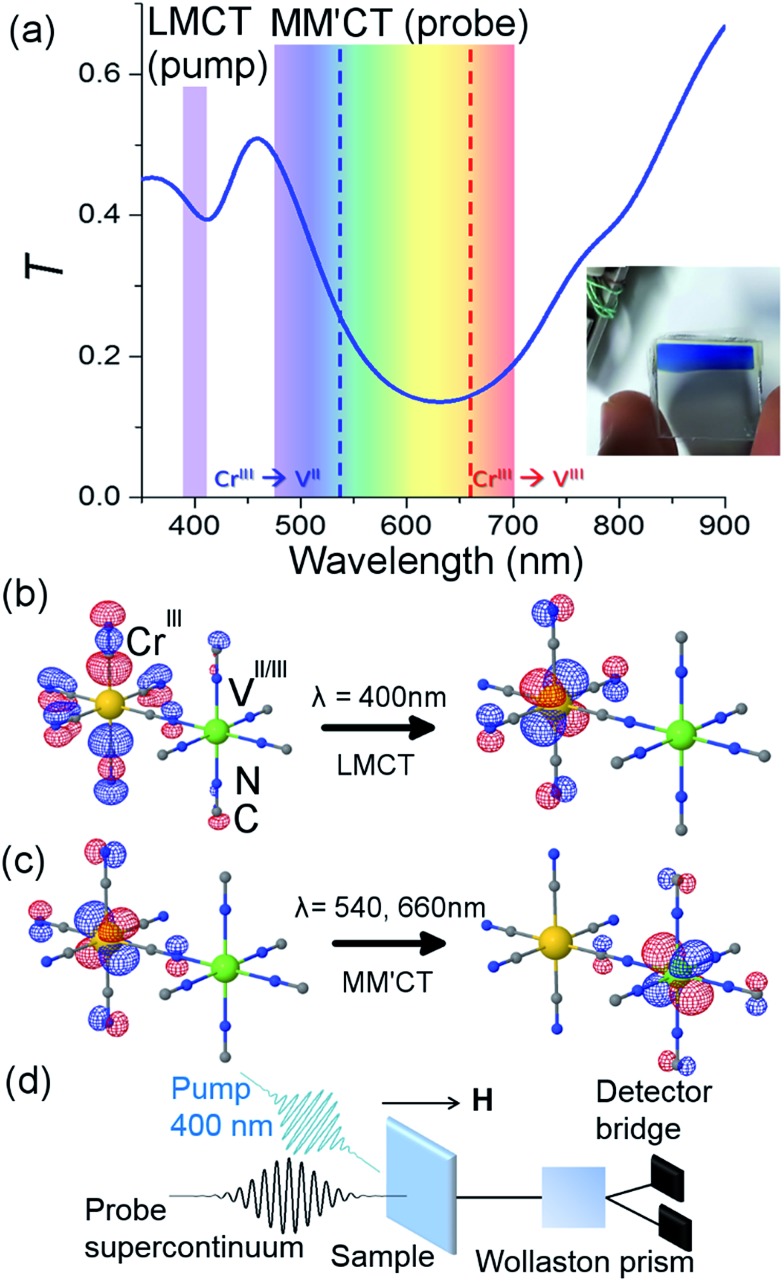
Orbital configuration and transmission associated with the ligand-to-metal and metal-to-metal charge transfer. (a) Static optical transmission spectrum of the film at room temperature. The pump wavelength is assigned to the ligand-to-metal charge transfer (LMCT) transition. The probe wavelengths span the visible part of the spectrum and measure changes related to the metal-to-metal charge-transfer (MM′CT) transitions for the two different V oxidation states present in the material. A photo of the film is shown in the inset. TD-DFT computations show (b) the LMCT transition and (c) the MM′CT transition. (d) Sketch of the femtosecond MO Faraday experimental setup.

The sample and a typical transmittance spectrum are shown in Fig. 1(a). The V^II/III^–Cr^III^ PBA is composed of Cr ions in their third oxidation state (Cr^III^, 3d^3^ electrons in the configuration t32ge0g) octahedrally surrounded by cyanide ligands (CN^–^) with the carbon end (grey spheres) towards the Cr ions (yellow spheres) and the V ions (green spheres) bound to the nitrogen (blue spheres) end of the ligands (see Fig. 1(b)). In the film, V is present in two different oxidation states, V^II^(t32ge0g) and V^III^(t22ge0g), and the corresponding ratio is determined by the electrochemical conditions used during deposition.^37^ The electrons are only partially localised on the metal ions and there is some orbital overlap between adjacent ions *via* the cyanide ligands. This leads to a coupling of the spins *via* the ligand bridge and the magnetic properties of the PBA therefore arise from the super-exchange interaction between the metal ions through the cyanide ligand (Fig. 1(b)). Due to the stoichiometry of the materials, vacant sites, and the presence of both V^II^ (*S* = 3/2) and V^III^ (*S* = 1) there is not a complete cancellation of the V spins with respect to the Cr^III^ spins (*S* = 3/2) and consequently the V–Cr PBA is a ferrimagnet.

We electrochemically synthesised thin films of the V^II/III^–Cr^III^ PBA on 3 mm thick fluorine-doped tin oxide (FTO) coated glass substrates under potentiostatic conditions as outlined in ref. 37 and 38. Aqueous solutions of VCl_3_ and K_3_[Cr(CN)_6_] from Sigma-Aldrich were used without further purification at concentrations of 15 and 10 mM and KCl was used as the electrolyte at a concentration of 0.5 M. The substrates were cleaned in an ultrasonic bath using three different solvents (clean substrates were critically important in order to produce films of good optical quality and thus reduce the amount of scattered pump light in the time-resolved experiments). A potential of –1.2 V w.r.t. a Pt pseudo-reference electrode was applied for 10 minutes, producing blue-coloured films, which showed transmittance spectra in accordance with the literature.^37,44^ Inductively coupled plasma optical emission spectrometry showed a Cr/V ratio of 0.89 and the IR spectrum showed an intense peak at 2106 cm^–1^ assigned to the asymmetric CN^–^ stretching frequency (ESI†). The V–Cr PBAs are air sensitive and so the electrochemistry was performed under a flow of N_2_. The films were rinsed with N_2_-bubbled H_2_O and allowed to dry under a flow of N_2_. Once dried, they were sealed with cyanoacrylate glue and a 0.18 mm thick glass microscope coverslip.

### Time-resolved configuration

The pump-probe MO configuration is sketched in Fig. 1(d). The laser system is an amplified titanium sapphire laser delivering 50 fs pulses at 5 kHz, with a central wavelength at 800 nm. Part of the beam is used to generate the pump pulses by frequency doubling (400 nm) in a β-barium borate crystal. The pump power was adjusted using a combination of a half waveplate and a polariser in order to achieve a pulse energy of 100 nJ. The beam was focused with a 25 cm achromatic lens to achieve a fluence of 0.5 mJ cm^–2^. At this pump energy, the samples were stable for *ca*. 10 min after which some degree of photodegradation was observed. For this reason, all experiments were performed on the same sample but at a different sample position for each measurement. The transmittance was checked before and after each measurement and because of the good sample homogeneity it was possible to measure at different spots with the same transmittance. The pump wavelength spectrally overlapped with the ligand-to-metal charge transfer (LMCT) UV bands, where an electron transfers from a CN^–^ ligand onto the Cr ion (Fig. 1(b)). Another part of the beam is used to generate a supercontinuum (*λ* = 480–690 nm) in a sapphire crystal by self-phase modulation. The supercontinuum is used to measure the time-dependent differential transmission (Δ*T*/*T*) and MO response (Faraday rotation, Δ*θ*_F_). A folded dispersive optical line allows for partial compensation of the chirp of the probe pulses. A variable slit in this dispersive line allows the narrower spectral probe wavelengths to be selected. In total eight wavelength-specific kinetics traces were recorded in the range of 480–690 nm with a 15 nm bandwidth. The fluence in the 15 nm spectral band was *ca.* a factor of 1000 lower than the pump energy. The pump and probe delay line is moved by a stepper motor. The overall pump and probe temporal resolution is ∼250 fs. The Faraday rotation is measured with a balanced polarisation bridge analysis. The signal-to-noise ratio in the transmission is minimised by an appropriate reference signal selected from the incoming probe beam. All signals are detected using a modulation and lock-in synchronous detection scheme.^4,5^ The temperature *T*_s_ of the sample is controlled with a cryostat and the magnetic field, applied perpendicular to the sample plane, is provided by a superconducting magnet.

### Computational methods

TD-DFT computations were carried out in order to give further support to the assignment of the optical transitions. Due to the complexity of the PBA system, we carried out the calculations for a single monomeric unit comprising one V with five CN^–^ ligands (with N pointing toward V), one Cr with five CN^–^ ligands (with C pointing toward Cr) and one bridging CN^–^ ligand (with N toward V and C toward Cr). Gaussian 09 (ref. 39) was employed to perform the TD-DFT calculations using the PBE0 hybrid functional.^40^ The calculations were performed at a fixed geometry and the 6-311G(d) basis set^41^ was used for V and Cr ions, and the 6-31G(d) basis set^42,43^ for C and N atoms. The symmetry of the monomeric unit was *C*_4V_. The calculations for the V^III^–Cr^III^ PBA showed two transitions with non-zero oscillator strengths in the UV/VIS spectrum, namely a LMCT from the CN^–^ ligand to the Cr t_2g_ orbital at 401 nm and a metal-to-metal charge-transfer (MM′CT) transition at 780 nm from the Cr t_2g_ to the V t_2g_ orbital. For the V^II^–Cr^III^ PBA system, the transitions were mixed. Here two degenerate LMCT transitions were identified at 324 nm and showed a mix of transfer from the CN^–^ ligand to both the Cr and V t_2g_ orbitals. The MM′CT transition was also mixed between transitions between t_2g_ orbitals from Cr to V and V to Cr and occurred at 572 nm. The red-shift of the MM′CT from lower to higher oxidation state of the V (572 and 780 nm, respectively) is in qualitative agreement with experiments.^37^

## Results and discussion

**Fig. 2:**
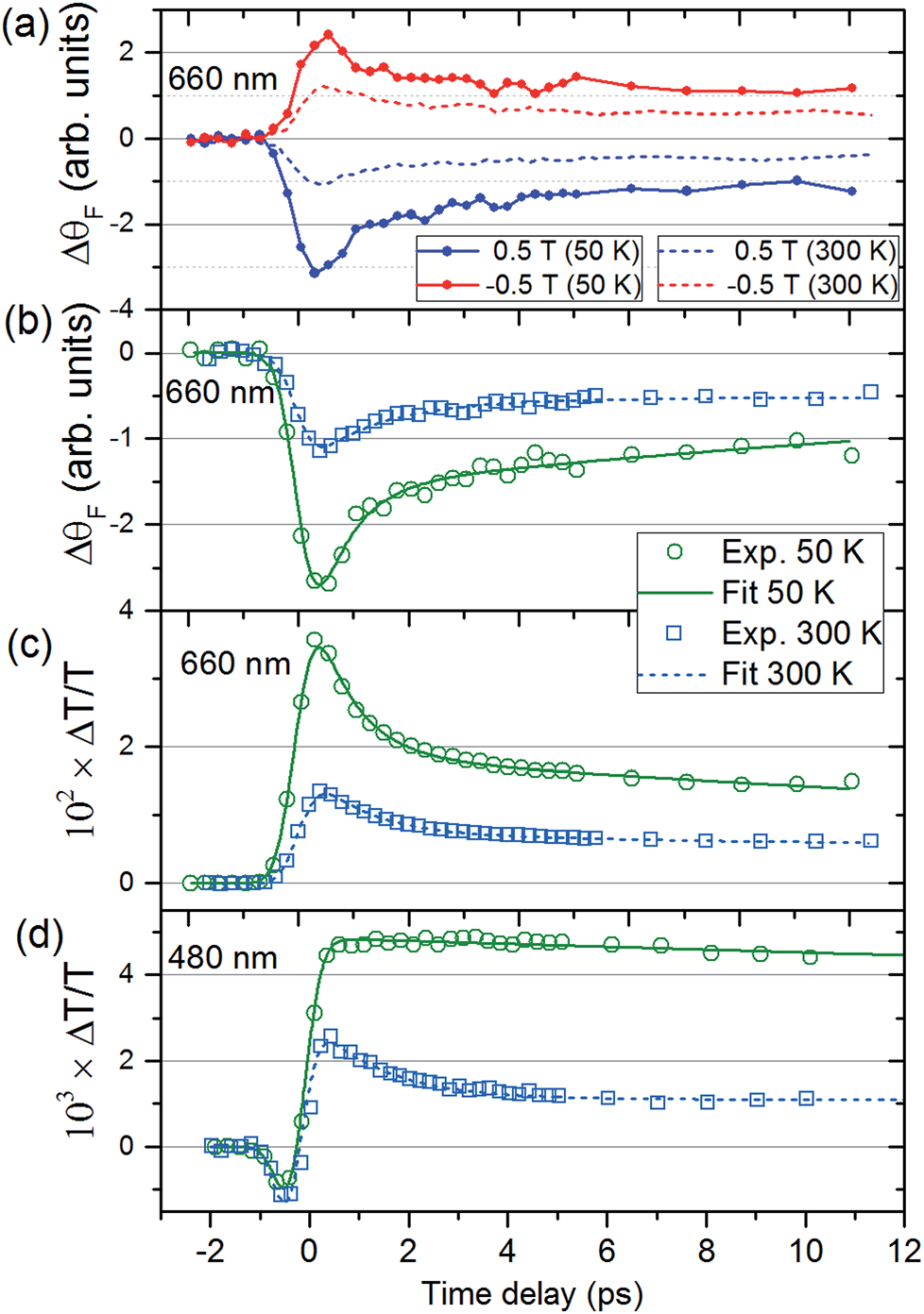
Time-dependent magneto-optical Faraday rotation and transmission of the V–Cr Prussian blue analogue film. (a) Dynamics of the magneto-optical signals for *H* = ±0.5 T, as a function of pump-probe delay for the probe wavelength of *λ* = 660 nm at *T*_s_ = 50 K (solid lines) and 300 K (dashed lines). (b) Difference between the MO Faraday signals in (a) together with fits. The corresponding transmission dynamics are shown in (c) for *λ* = 660 nm. Transmission (d) for *λ* = 480 nm for the two temperatures.

Fig. 2(a) shows Δ*θ*_F_ at *T*_s_ = 50 and 300 K for *λ* = 660 nm. The signals are recorded for antiparallel magnetic field directions, perpendicular to the sample plane (*H* = ±0.5 T), and the difference between the two signals is shown in Fig. 2(b). As is seen in Fig. 2(a) and (b), a change in the MO signal occurs on a sub-picosecond timescale. It should be noted that Δ*θ*_F_ has not been normalised for the static Faraday signal *θ*_F_. The dynamics are fitted with a causal exponential decay, taking into account the Gaussian temporal profile of the pump laser. After a fast rising part, relaxation occurs with the time-constants *τ*_Δ*θ*_ (50 K) = 0.64 ps and *τ*_Δ*θ*_ (300 K) = 1.31 ps at *λ* = 660 nm. The corresponding dynamics of the transmission are displayed in Fig. 2(c) with similar time constants of *τ*_Δ*T*_ (50 K) = 0.76 ps and *τ*_Δ*T*_ (300 K) = 1.05 ps. The fast decay reaches a plateau that slowly decays over several hundreds of picoseconds (shown in ESI†). For *λ* = 480 nm (Fig. 2(d)) the dynamics of both Δ*θ*_F_ and Δ*T*/*T* are different at 50 K, where only the plateau is observed. For this wavelength, a negative signal around zero time delay is also observed at both temperatures.

**Fig. 3:**
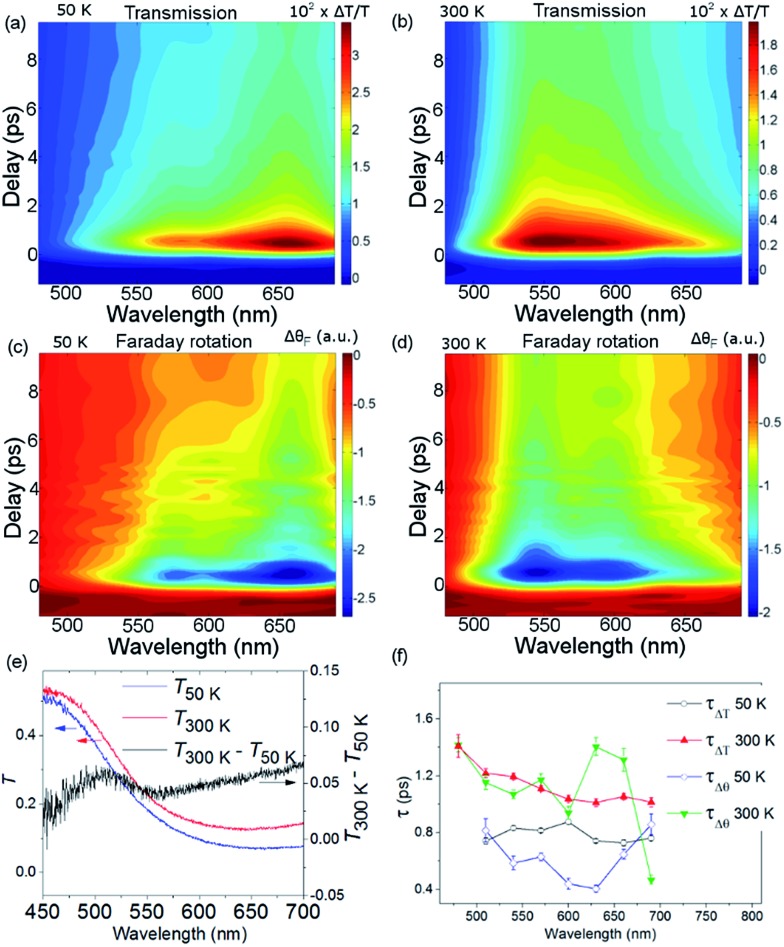
Spectro-temporal dynamics of the transmission and magneto-optical Faraday response for *T*_s_ = 50 and 300 K. (a) and (b) show Δ*T*/*T* and (c) and (d) show Δ*θ*_F_ as a function of wavelength and time delay. (e) The static transmission spectra as a function of temperature. (f) Fitted time constants from the data in (a)–(d).

Fig. 3, obtained by interpolation of wavelength-specific kinetic traces, summarises the spectro-temporal dynamics of Δ*T*/*T* and Δ*θ*_F_ over the whole probe supercontinuum for *T*_s_ = 50 and 300 K. The maxima of the dynamical spectra are shifted for the two temperatures. The temperature is clearly important for the dynamics after pumping at the LMCT and can be seen to also play a role in the static transmittance spectra of non-photoexcited films (Fig. 3(e)). Fig. 3(a)–(d) show that the fast initial decay reaches a plateau (although for *λ* = 480 nm at 50 K, the signal immediately reaches the plateau). This is shown in detail in Fig. 2(b) and (c). Fig. 3(f) shows the fitted time constants from the wavelength-specific kinetic traces for the decays over the probe spectrum. The decays are faster at *T*_s_ = 50 K than at 300 K for both *τ*_Δ*θ*_ and *τ*_Δ*T*_.

**Fig. 4:**
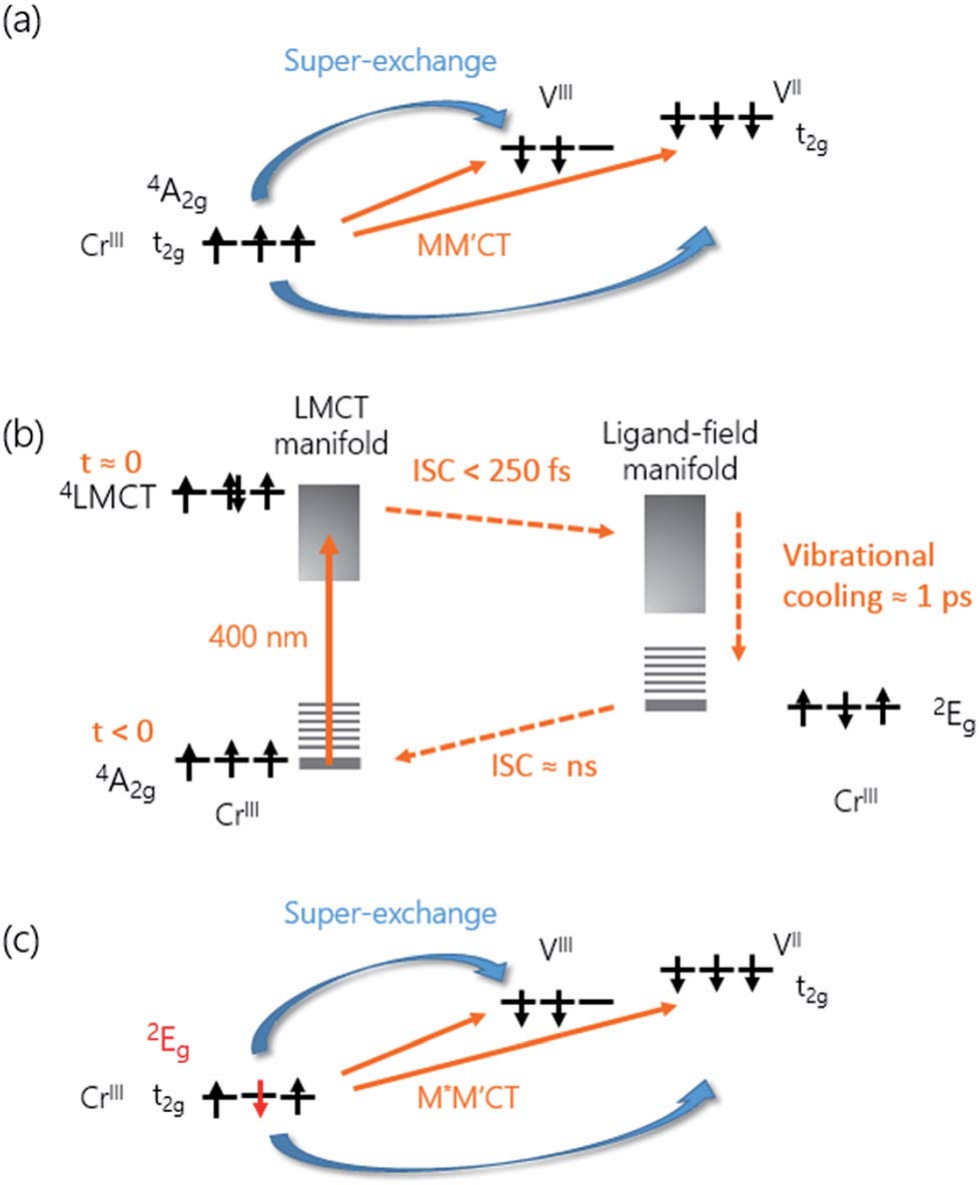
Charge and spin dynamics model. (a) In the ground-state, the MM′CT can occur from the Cr^III^ ions to the V^II^ and V^III^ ions. (b) The pump laser excites an electron from a CN^–^ ligand onto the Cr ion (LMCT). The populated ^4^LMCT state quickly decays to the LF manifold and the vibrationally excited ^2^E state is formed in less than 250 fs. Subsequent vibrational cooling in this state takes place on a *ca.* 1 ps timescale. The ^2^E state eventually decays back to the ground state *via* ISC on a ns timescale. (c) The ^2^E state has a different spin configuration compared to the ^4^A_2_ ground state. The associated change in the super-exchange interaction affects the MO signal of the M*M′CT state.

The overall spin and charge dynamics after the LMCT to Cr are interpreted by considering that the probe pulses overlap with the MM′CT band (Fig. 1(c) and 4(a)). Because of the different oxidation states of the V ions, more energy is required to transfer an electron from a Cr ion to a V^II^ site than to a V^III^ site due to the Coulomb repulsion. This difference results in the splitting of the MM′CT band into two peaks in the transmittance spectrum at 660 nm (Cr^III^ → V^III^) and at 540 nm (Cr^III^ → V^II^), which is barely seen as a shoulder in the transmittance spectrum shown in Fig. 1(a). Such a peak was observed at 550 nm by Garde *et al.* for V^II^–Cr^III^ PBA molecules in suspension who also assigned it to the MM′CT band.^44^ The static Faraday ellipticity spectrum reported by Ohkoshi *et al.* ^37^ gives further support for the two types of charge-transfer. Indeed, in their PBA films the predominance of V^II^ ions strongly reduces the resonance at 660 nm associated with the V^III^ ions.

The increase in transmission of the MM′CT states and subsequent fast decay has to be interpreted by considering that we are pumping at the LMCT transition. In contrast to metallic materials, where the excitation energy is quickly redistributed among all electrons on a femtosecond timescale, the electron dynamics in transition metal complexes depend on the pump wavelength which may excite transitions that are (i) localised ligand–ligand or metal-centred ligand-field (LF) transitions or (ii) partially delocalised LMCT/MLCT or MM′CT transitions. Ultrafast relaxation dynamics after fs excitation in Cr^III^(acac)_3_ (acac = deprotonated monoanion of acetylacetone) complexes in solution at both LF and LMCT pumping have been extensively studied by Juban and McCusker.^32,45^ They found that the excited ^4^LMCT state quickly decays *via* intersystem crossing (ISC) to the ^2^E state of the Cr ion with a 50 fs time constant. Subsequent decay kinetics of the signal on a 1.1 ps timescale was attributed to vibrational cooling in the ^2^E state. The ^2^E state eventually decays back *via* ISC to the ^4^A_2_ ground state on a ns timescale. ISC on timescales shorter than 100 fs after MLCT excitation is known to occur in Fe^II^ SCO complexes in solution^23^ and it has been reported that similar dynamics, localised on the Fe sites in the lattice, can be observed in SCO crystals.^21,25^ It is therefore plausible that the decay processes described by Juban and McCusker^32,45^ are applicable to the dynamics of the Cr ions in the V^II/III^–Cr^III^ PBA lattice. It should be noted that for the shortest wavelength (*λ* = 480 nm, Fig. 2(d)) we observe a very fast transient decrease of the transmission, which we attribute to an excited-state absorption (ESA) from the ^4^LMCT state. The subsequent fast decay of the ESA at 480 nm (180 ± 30 fs at 50 K and 110 ± 10 fs at 300 K, both time constants shorter than the experimental time resolution), which occurs at the very beginning of the pump-probe Δ*T*/*T* signal, further supports the short life-time of the ^4^LMCT state. The subsequent formation of the excited ^2^E state corresponds to a spin flip of one of the electrons in a t_2g_ orbital and for this reason we will hereafter name the metal-to-metal charge-transfer process M*M′CT instead of MM′CT, where M* indicates an excited state of the Cr ion. The new spin configuration on the Cr site will affect the M*M′CT transition leading to a reduction in the MM′CT absorption causing the increase in the transmission that we observe experimentally. The vibrational cooling in the ^2^E state is responsible for the ∼0.8 ps at 50 K and ∼1.1 ps at 300 K decay of the transient transmission that we observe (Fig. 4(b)). We do not observe any ESA from the ^2^E state,^45^ presumably because changes to the visible spectrum are completely dominated by the much stronger M*M′CT transition.

The propensity to optically transfer to either V^II^ or V^III^ from the excited-state potential, and the corresponding timescale for which this occurs, depends on the sample temperature, as observed in Fig. 3(a), (b) and (f). The temperature dependence of the M*M′CT absorption band should therefore be different from the temperature dependence of the ground-state MM′CT band as displayed in the static spectrum of Fig. 3(e). The above interpretation of a temperature-dependent decay pathway after the LMCT excitation is further sustained by the results of Bozdag *et al.* ^46^ who identified a hidden metastable state that caused a decrease in the magnetisation after illuminating a sample of V–Cr PBA for 60 h at the LMCT transition at 10 K (*λ* = 350 nm). In their results, the metastable state survives heating up to 250 K and disappears at higher temperatures, indicating the efficient role played by thermal excitations in PBAs.

Let us now focus on the differential magneto-optical Faraday signal Δ*θ*_F_ measured at temperatures *T*_s_ = 50 and 300 K. Electronic optical transitions that affect exchange-coupled electrons give rise to a MO signal whose magnitude and sign depend on the nature of the transition.^31^ Ohkoshi *et al.* have shown that the MO signal from the MM′CT transition in V–Cr PBA is proportional to the magnetisation.^37^ This arises because the spins on the Cr ions are connected to the spins on the V ions *via* the super-exchange interaction. In contrast, there is no static MO signal from the LMCT transition,^37^ which is probably due to the fully occupied orbital of the ligand and so there is no exchange interaction between the electrons on the ligand and the Cr ion. In our experiments, we observe similar decay constants as for Δ*T*/*T*, which is shown in Fig. 3(f). After the fast ISC to the ^2^E state on the Cr ion, the new spin configuration is changed from *S* = 3/2 to *S* = 1/2 (Fig. 4(c)). The local change in spin configuration modifies the super-exchange interaction between the Cr and V ions and therefore gives rise to the change in MO signal for the M*M′CT transition. The time-resolved MO signal can therefore detect changes in the strength of the super-exchange interaction on a sub-ps timescale.

Besides from the large range of studies on SCO compounds mentioned previously, where fast ISC occurs accompanied by structural changes, other molecular magnetic systems also display fast dynamics. For example, fast sub-50 fs three-state dynamics, involving partly spin-allowed steps, have been observed in breathing crystals using transient absorption.^12^ In Prussian blue, both ultrafast back-electron transfer and trapping of charge-transfer (CT) states occur after exciting at the MM′CT transition.^47,48^ This has also been observed in related dinuclear cyano-bridged mixed-valence systems.^49^ Trapping of long-lived CT states (ns) in the photomagnetic Co^II^–Fe^III^ PBA was also observed after both MM′CT and LMCT pumping.^50^

## Conclusions

In conclusion, we have observed the ultrafast dynamics of charge and spin transfer in the molecule-based magnet V–Cr PBA at room temperature and 50 K. It has been carried out by performing time-resolved femtosecond transmission and magneto-optical Faraday measurements with frequency non-degenerate pumping (400 nm) and probing (super-continuum in the visible). We show that upon exciting the ligand-to-metal charge-transfer transition at 400 nm, the ^2^E state on the Cr sites is populated in less than 250 fs, resulting in an increase in the transmission associated with the M*M′CT transition. Vibrational cooling in the ^2^E state occurs with a time constant of 0.78 ± 0.05 ps at 50 K and 1.1 ± 0.1 ps at 300 K. Correspondingly the time-dependent MO Faraday signal follows the same dynamics and the associated change in spin-configuration of the ^2^E state is observable in the MO signal of the M*M′CT transition. The results show that this method can be used to directly observe changes to spin configurations, and therefore the exchange interaction, on a fs timescale in magnetic molecular materials. The signature from both V oxidation states implies that the present approach of studying site-specific dynamics using ultrafast laser spectroscopy together with time-resolved magneto-optics is a powerful and underexplored technique for the field of molecular magnetism, especially when selective pumping and broad-band spectral probing are employed. This in turn will allow for new chemistry to be developed in the process of optimising magneto-optical properties and spin switching rates by chemically tuning the molecular properties. A sudden change in spin configuration can lead to a large structural change, as in the case of spin-crossover materials, which typically leads to vibrational dynamics involving stretching and bending modes.^21,23,25^ The proposed method here is fast enough to follow the vibrational dynamics of magneto-structural correlations by simultaneously recording the transmission and MO signals. Faraday MO techniques can therefore provide an attractive alternative approach to directly probe the spin dynamics in molecule-based magnets, single-molecule magnets, and spin-crossover materials. These studies show the large potential of femtomagnetism for studying and monitoring molecular magnets.
