# Supplementary material for: Directly probing spin dynamics in a molecular magnet with femtosecond time-resolution
Source: Chem Sci. 2016 Aug 1;7(12):7061–7. doi: 10.1039/c6sc01105e (PMC5355827; doi:10.1039/c6sc01105e)
Supplement: Supplementary file 1 [file SC-007-C6SC01105E-s001.pdf]

Electronic Supplementary Material (ESI) for Chemical Science.
This journal is © The Royal Society of Chemistry 2016

Supporting information

Directly probing spin dynamics in a molecular magnet with femtosecond time-resolution

J. O. Johansson,*[a] J.-W. Kim,[b] E. Allwright,[a] D. M. Rogers,[a] N. Robertson[a] and J.-Y. Bigot*[b]

[a.] *EaStCHEM, School of Chemistry, University of Edinburgh, David Brewster Road, EH9 3FJ, UK. Email: olof.johansson@ed.ac.uk*
[b.] *Institut de Physique et Chimie des Matériaux de Strasbourg (IPCMS), UMR 7504, CNRS, Université de Strasbourg, BP 43, 23 rue du Loess, 67034 Strasbourg Cedex 02, France. Email: jean-yves.bigot@ipcms.unistra.fr*

S1 – Materials

The sample was exposed to air and the IR spectrum was quickly measured in an attenuated total reflectance (ATR) spectrometer and is shown in the figure below. The peak at 2106 cm^{-1} is assigned to the VCr PBA, as reported previously by several groups[1–3]. During the measurement, the sample was partially oxides in air as confirmed by the presence of the peak at 2164 cm^{-1}, which has previously been assigned to the Cr^{III}-CN-$V^{IV}O$ in ref. [1].

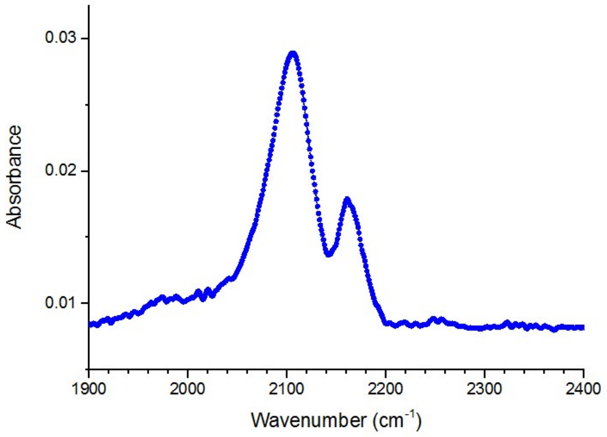

Figure 1: ATR-IR spectrum of the VCr PBA film.

S2 - Decay up to 40 ps (660 nm).

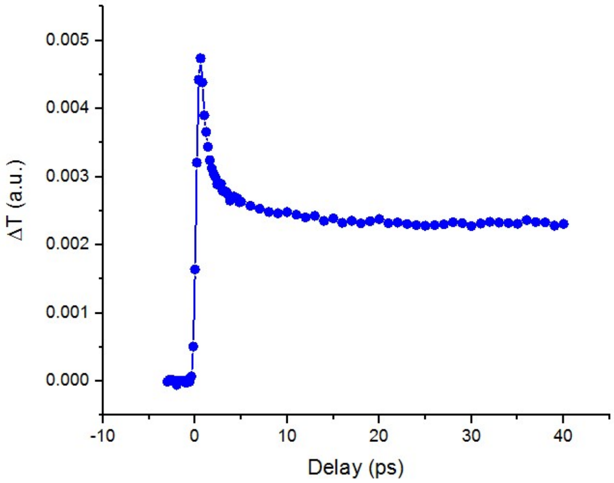

Figure 2: Transient transmission data at 660 nm of a VCr PBA film after exciting at 400 nm. The sample temperature was 50 K.

S3 – Additional comments on fitting procedures

For each wavelength-specific kinetic trace, the time-zero delay is set using the optical Kerr effect signal from the glass substrate. A finer time-zero is subsequently selected by deconvoluting the kinetic traces with the instrument response function. A large part of the chirp compensation of the continuum is obtained by the double pass prisms compressor. The residual group velocity dispersion has been characterized[4] by two photon absorption in a thin semiconductor crystal with a large band gap (ZnS). It is of the order of 850 fs in the entire spectral range and less than 150 fs in the range 500–650 nm.

S3 –Wavelength-specific Faraday rotation and transient transmission data with fits.

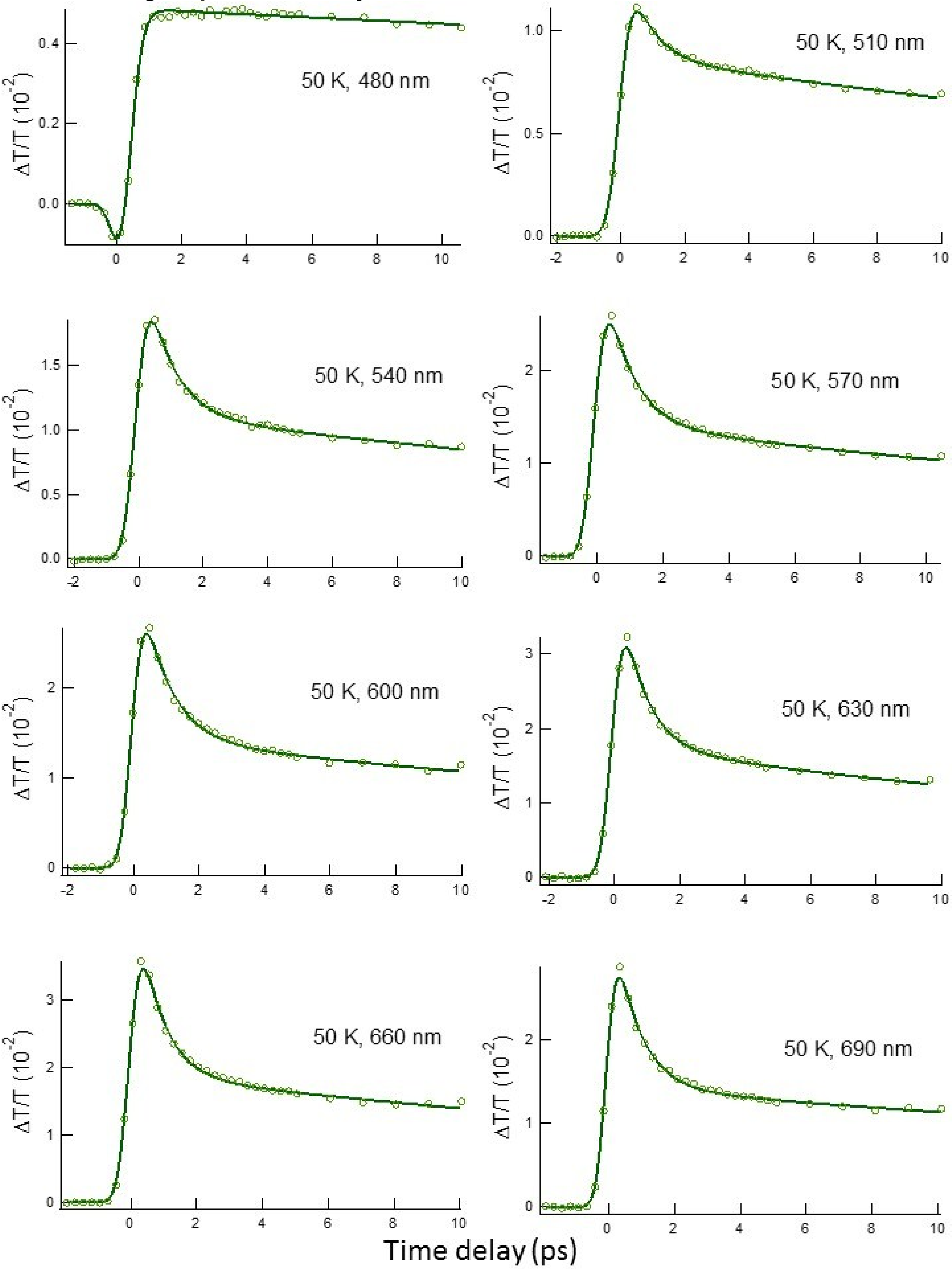

Figure 3 Transient transmission data with fits for 50 K.

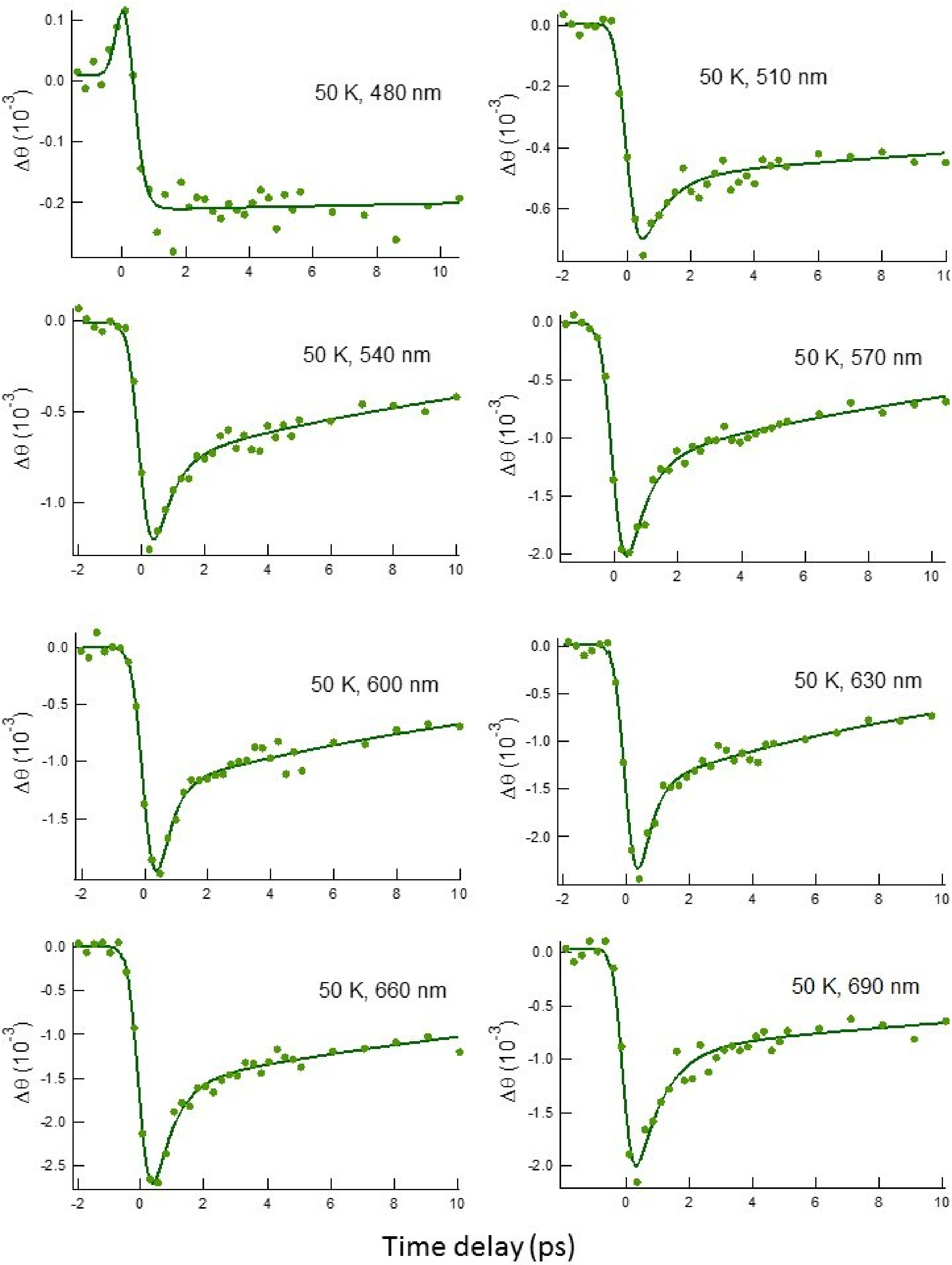

Figure 4 Faraday rotation data with fits for 50 K.

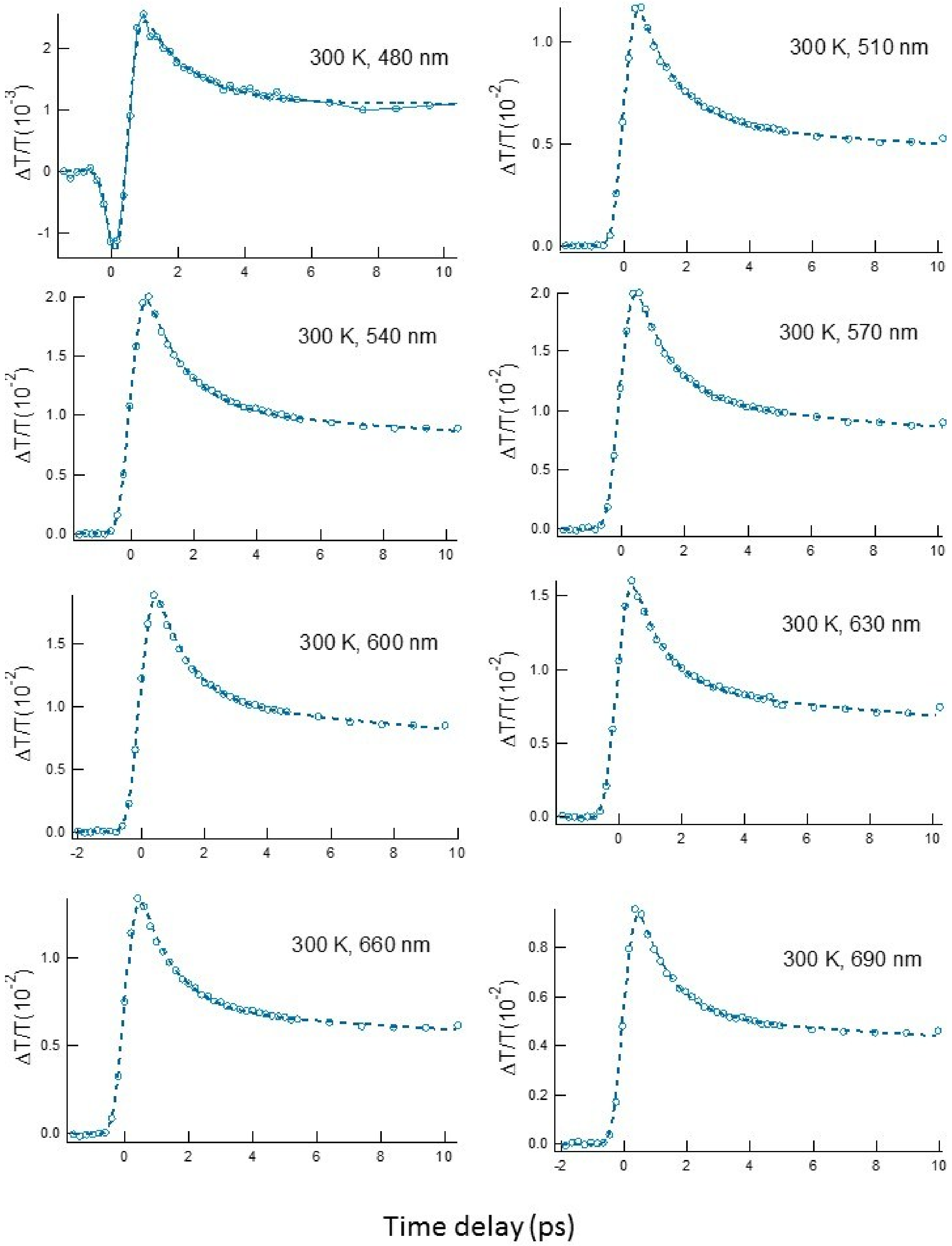

Figure 5 Transient transmission data with fits for 300 K.

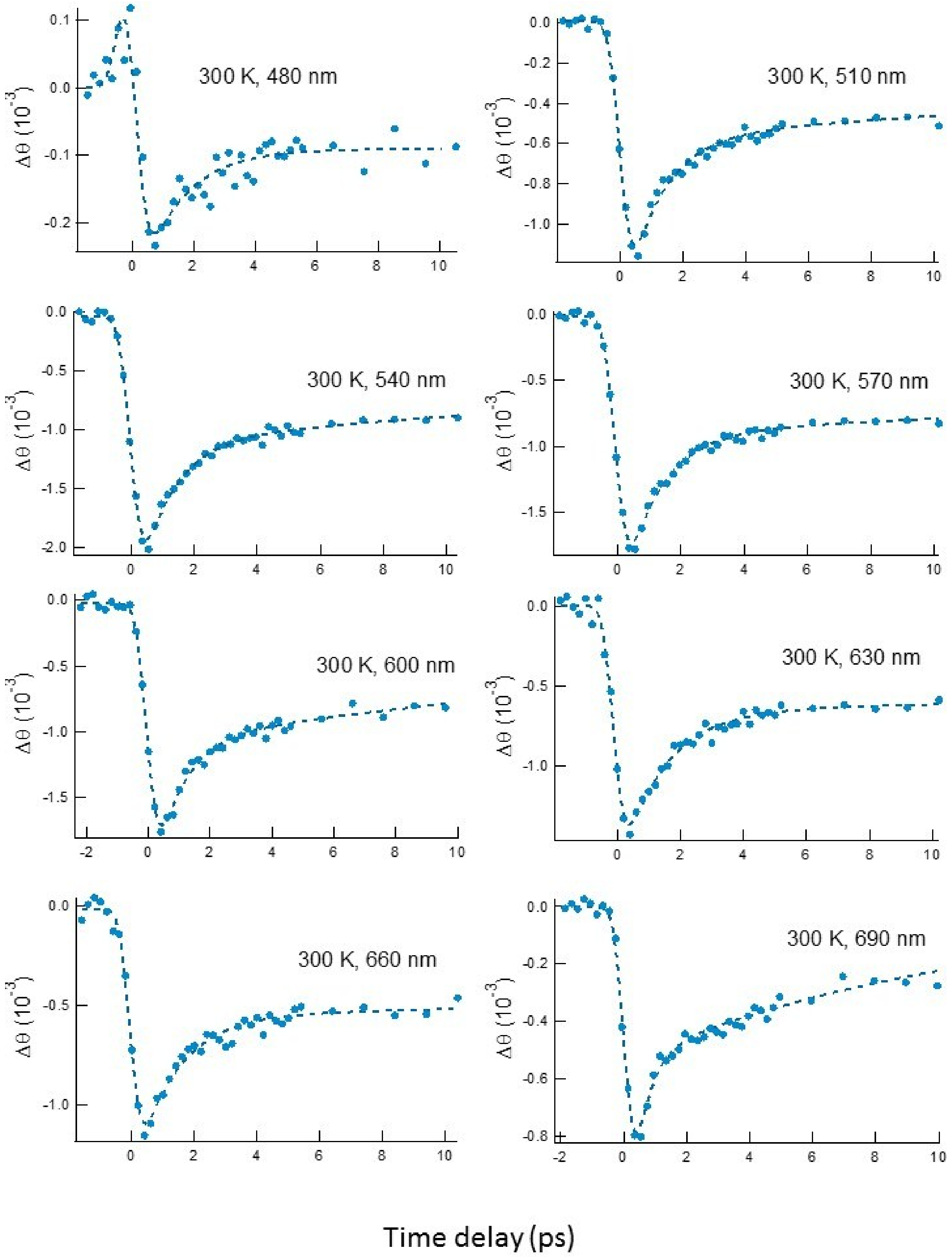

Figure 6 Faraday rotation data with fits for 300 K.

S4 – References (for supporting information)

1 S. Ferlay, T. Mallah, R. Ouahès, P. Veillet and M. Verdaguer, *Nature*, 1995, **378**, 701–703.

2 Ø. Hatlevik, W. E. Buschmann, J. Zhang, J. L. Manson and J. S. Miller, *Adv. Mater.*, 1999, **11**, 914–918.

3 S. Ohkoshi, M. Mizuno, G. Hung and K. Hashimoto, *J. Phys. Chem. B*, 2000, **104**, 9365–9367.

4 J.-Y. Bigot, L. Guidoni, E. Beaurepaire and P. Saeta, *Phys. Rev. Lett.*, 2004, **93**, 077401.
